# The anti-apoptotic Bcl-2 family protein A1/Bfl-1 regulates neutrophil survival and homeostasis and is controlled via PI3K and JAK/STAT signaling

**DOI:** 10.1038/cddis.2016.23

**Published:** 2016-02-18

**Authors:** J Vier, M Groth, M Sochalska, S Kirschnek

**Affiliations:** 1 Institute of Medical Microbiology and Hygiene, Department of Medical Microbiology and Hygiene, University Medical Centre Freiburg, Freiburg, Germany; 2 Division of Developmental Immunology, Biocenter, Medical University Innsbruck, Innsbruck, Austria

## Abstract

Neutrophil granulocytes are innate effector cells of the first line of defense against pyogenic bacteria. Neutrophil lifespan is short, is prolonged by pro-inflammatory stimuli, controls functionality of the cells and can determine tissue damage. Experimental analysis of primary neutrophils is difficult because of their short lifespan and lack of possibilities of genetic manipulation. The Hoxb8 system of neutrophil differentiation from immortalized progenitor cells offers the advantage of unlimited production of neutrophils *in vitro* as well as easy genetic modification. We here use this system to analyze the role of the poorly characterized anti-apoptotic B-cell lymphoma protein 2 (Bcl-2) family member A1/Bfl-1 (Bcl-2-related protein A1) for survival and homeostasis of neutrophils and of neutrophil progenitors. Low constitutive mRNA and protein expression of A1 was detected, while A1 was transiently upregulated early during differentiation. Pro-inflammatory stimuli caused strong, mainly transcriptional, A1 upregulation, in contrast to posttranscriptional regulation of Mcl-1 (induced myeloid leukemia cell differentiation protein). Inhibitor studies showed that phosphoinositide-3 kinase (PI3K)/Akt and Janus kinase (JAK)/signal transducer and activator of transcription (STAT) is required for A1 expression and survival of progenitors and mature neutrophils. ShRNA-mediated constitutive A1 knockdown (KD) impaired maintenance of progenitors. ShRNA experiments further showed that A1 was required early during neutrophil differentiation as well as in mature neutrophils upon pro-inflammatory stimulation. Our data further indicate differential regulation of the two anti-apoptotic proteins A1 and Mcl-1. Relevant findings were confirmed in primary human neutrophils. Our data indicate that A1, in addition to the well-established Mcl-1, substantially contributes to neutrophil survival and homeostasis. A1 may thus be a promising target for anti-inflammatory therapy.

Neutrophil granulocytes (neutrophils) belong to the first line of defense of the innate immune system and have a crucial role in the antimicrobial response against extracellular bacteria. Neutrophils are constantly released from the bone marrow into the blood and are recruited to sites of infection. Neutrophils have also been implicated in the response against intracellular bacteria and viruses and in tumorigenesis.^1^ Prolonged neutrophil activity can be detrimental. Inhibition of neutrophil apoptosis led to increased tissue damage in an experimental mouse model of bacterial meningitis,^2^ whereas the promotion of inflammatory cell apoptosis enhanced resolution of inflammation.^2, 3^

Neutrophils have a lifespan of few hours, which is regulated by apoptosis.^4^ Pro-inflammatory stimuli such as cytokines or bacterial components can prolong neutrophil lifespan.^4, 5^ Apoptosis also terminates neutrophil activity and regulates neutrophil-induced inflammation.^2, 6^

Neutrophil apoptosis is regulated by the mitochondrial pathway of apoptosis and the family of B-cell lymphoma protein 2 (Bcl-2)-like proteins. Within the anti-apoptotic Bcl-2-like subfamily, Mcl-1 (induced myeloid leukemia cell differentiation protein) seems to be the most important survival factor for hematopoietic cells. Conditional deletion of Mcl-1 in the hematopoietic/myeloid compartment revealed a crucial role of Mcl-1 for survival of stem cells,^7^ cells of the lymphocyte lineage^8^ and the neutrophil- but not monocyte/macrophage lineage.^9^ In contrast, the role of A1 for neutrophil development and homeostasis is much less clear. A1 was first described as hematopoietic tissue-specific, granulocyte-macrophage colony-stimulating factor (GM-CSF)-regulated gene.^10, 11^ Upregulation of A1 mRNA was observed in neutrophils upon contact with intracellular pathogens.^12, 13, 14^ Differential effects on expression of Mcl-1 and A1 were shown in tumor necrosis factor-stimulated neutrophils.^15^ Until recently, analysis of A1 was hindered by the lack of sensitive antibodies. Genomic deletion of A1 in mice is complicated by the existence of four genes (A1-a,-b,-c (a pseudogene),-d), in the mouse.^16^ Knockout of murine A1-a resulted in enhanced spontaneous neutrophil apoptosis.^17^ An A1 shRNA knockdown (KD) mouse approach was established by Ottina *et al.*^18^ targeting all functional genes. This constitutive A1 KD affected lymphoid and myeloid cells by reducing the colony formation potential of granulocytic precursors and their accumulation *in vivo.*^18, 19^ Nevertheless, the role of A1 in neutrophil development and homeostasis and its regulation in the neutrophil lineage is not well understood.

Major experimental drawbacks of neutrophil work are lacking possibilities of genetic modification in human cells and limited cell numbers in mice. These problems can be overcome using a model system of differentiation of murine myeloid cells from bone marrow progenitors.^20^ Conditional estrogen-regulated activation of the transcription factor Hoxb8 allows the establishment of progenitor lines committed to the neutrophil lineage in the presence of specific cytokines. This system enables large-scale production of mature neutrophils *in vitro*, but is also amenable to genetic modification. Progenitors express active Hoxb8, which keeps them undifferentiated akin to leukemic cells. Although this must be kept in mind, the study of progenitors stills adds information on regulation of cellular processes in cells having a maturation state similar to pro-myelocytes.^20^ When Hoxb8 is turned off, the cells quickly start differentiation into neutrophils, permitting analysis of gene regulation during this process, and finally the analysis of cells indistinguishable from mature neutrophils.^20, 21^

Here we analyzed the expression and regulation of A1 in progenitors, differentiating neutrophils and mature cells at steady-state and upon pro-inflammatory stimulation. We found strong regulation of A1 mRNA and protein expression, modulated mainly by phosphoinositide-3 kinase (PI3K)/Akt and Janus kinase (JAK)/signal transducer and activator of transcription (STAT) pathways, and less by extracellular signal-regulated kinase (ERK). Both A1 KD or kinase inhibition sensitized neutrophils and progenitors to apoptosis, indicating that A1 may be a promising target for anti-inflammatory therapy.

## Results

### Expression of A1 in differentiated neutrophils is dynamic and transcriptionally regulated, whereas Mcl-1 is mainly posttranscriptionally regulated

Progenitors committed to the neutrophil lineage were established from mouse bone marrow.^20^ Progenitors were induced to undergo neutrophil differentiation by estrogen withdrawal for 4 days, resulting in mature cells similar to primary bone marrow-sorted neutrophils. Characterization of differentiated Hoxb8 neutrophils on the basis of cellular and nuclear morphology, regulation of surface markers as well as functionality has been described in detail by us and others.^2, 20, 21, 22^ Typical differentiation-associated cellular and nuclear changes, like doughnut-shaped segmentation of nuclei, upregulation of Gr-1, CD11b and CXCR2, downregulation of c-kit, are illustrated in Supplementary Figure S2. Expression of A1 was analyzed at the protein (using a new antibody of enhanced sensitivity)^23^ and mRNA level in mature neutrophils.

**Figure 1:**
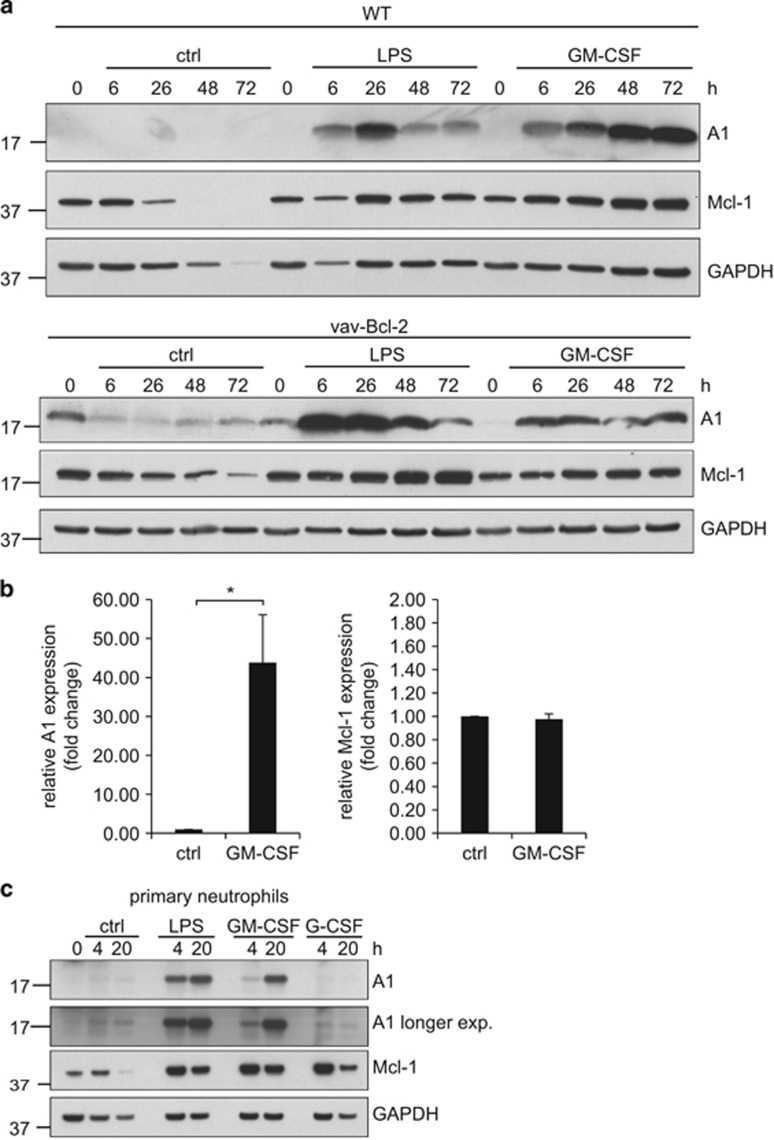
A1 expression at the mRNA and protein levels in differentiated neutrophils. (**a**) Protein expression levels of A1 and Mcl-1 in day 4 differentiated neutrophils analyzed by immunoblotting. Day 4 differentiated Hox8 wt (top) or vav-Bcl-2 transgenic (bottom) neutrophils (5 × 10^6^/5 ml per sample) were stimulated with LPS or GM-SCF, respectively, for the indicated time periods. Cells were lysed by boiling in Laemmli buffer, and equivalents of 1 × 10^6^ cells per lane were separated by SDS-PAGE, transferred onto nitrocellulose membrane and subjected to immunoblot analysis for A1 and Mcl-1. GAPDH served as a loading control. The relatively high level of A1 in Bcl-2-transgenic cells is not a consistent feature of these cells but rather illustrated the level of variation in a number of experiments. Data are representative for three (wt cells) or two (vav-Bcl-2-tg cells) independent experiments. (**b**) A1 mRNA levels in differentiated neutrophils. Day 4 differentiated Hoxb8 neutrophils (5 × 10^6^ per sample in total) were stimulated with GM-CSF for 4 h, harvested and total RNA was extracted and reverse transcribed. mRNA levels of A1 were quantified by real-time qRT-PCR on a Light Cycler 2 instrument. Expression is shown as fold change normalized to *β*-actin levels. Data represent mean/S.E.M. of six independent experiments (A1) or three independent experiments (Mcl-1). *P≤0.05 (paired Student's *t*-test). (**c**) Protein expression levels of A1 and Mcl-1 in primary mouse bone marrow-sorted neutrophils. Mouse neutrophils sorted by positive selection from bone marrow of C57BL/6 wt mice were stimulated with GM-CSF, G-CSF or LPS for the indicated time periods. After harvest, cells were lysed by boiling in Laemmli buffer and equivalents of 1 × 10^6^ cells per sample were separated by SDS-PAGE, transferred onto nitrocellulose membrane and subjected to immunoblotting against A1 and Mcl-1. GAPDH served as a loading control. Data are representative of three independent experiments

Weak expression of A1 protein was seen in resting differentiated cells. A1 protein was strongly upregulated by pro-inflammatory stimuli of host and microbial origin (GM-CSF, lipopolysaccharide (LPS); Figure 1a). Induction of A1 protein upon LPS stimulation was noticeable within the first hour in primary murine neutrophils (Supplementary Figure S3A), and similarly in Hoxb8 neutrophils (data not shown). LPS-induced A1 expression peaked at day 1 and then declined, whereas GM-CSF induced A1 more slowly but maintained expression over 3 days (Figure 1a). A1 expression was similarly induced by both 1% cell culture supernatant from GM-CSF-producing B16 cells and recombinant murine GM-CSF (10 ng/ml) (Supplementary Figure S1A) and correlated well with the strong GM-CSF-mediated apoptosis protection (Supplementary Figures S1B and C). One percent GM-CSF supernatant thus showed effects comparable to recombinant murine GM-CSF (10 ng/ml) (Supplementary Figure S1) and was used in the majority of experiments. LPS stimulation was able to prolong neutrophil survival, but to a lesser extent than GM-CSF (Supplementary Figures S1B and C).

To exclude that decline in A1 protein expression reflected protein loss because of spontaneous apoptosis, Bcl-2-overexpressing Hoxb8 cells derived from vav-Bcl-2-transgenic mice overexpressing human Bcl-2 in the hematopoetic compartment^24^ were analyzed. Vav-Bcl-2-transgenic differentiated Hoxb8 neutrophils as well as primary neutrophils are efficiently protected against spontaneous apoptosis for at least 3 days, as previously shown.^2, 21, 25^ Supplementary Figures S1B and C present additional evidence for efficient protection by Bcl-2. Bcl-2-overexpressing cells showed A1 protein expression comparable to wild type (wt; Figure 1a). In addition, induction of A1 protein by LPS and GM-CSF was confirmed in primary mouse neutrophils (Figure 1c). Slight apoptosis protection by LPS in primary neutrophils was visible by 20 h but this did not reach statistical significance, whereas GM-CSF treatment substantially protected against cell death (Supplementary Figure S3B).

A1 mRNA analysis revealed strong transcriptional regulation of A1 with >20-fold induction by GM-CSF in differentiated Hoxb8 neutrophils (Figure 1b), in accordance with previous results.^26^

Mcl-1 is known to be important for regulation of neutrophil survival, and A1 and Mcl-1 are believed to have similar molecular function.^27^ We therefore tested Mcl-1 regulation in neutrophils in parallel. Mcl-1 protein expression was easily detectable in freshly prepared cells and declined over time. LPS and GM-CSF maintained Mcl-1 protein expression and weakly induced additional Mcl-1 expression although this was much less prominent than for A1 (Figure 1a). Interestingly, granulocyte-colony-stimulating factor (G-CSF) stimulation in primary neutrophils led to early upregulation of Mcl-1 but not A1 protein (Figure 1c). Although the data indicate that A1 is strongly regulated at the transcriptional level, no indication of transcriptional regulation of Mcl-1 by GM-CSF was found (Figure 1b, right). Pro-inflammatory stimuli thus increased expression of both anti-apoptotic proteins, with considerably stronger induction of A1. Transcriptional regulation was, however, seen exclusively for A1.

In order to investigate the potential impact of further anti-apoptotic proteins besides Mcl-1 and A1, notably Bcl-2 and Bcl-2-like protein 1 isoform 1 (Bcl-x_L_), GM-CSF-stimulated cells were treated with the Bcl-2/Bcl-x_L_ inhibitor ABT-737,^28^ which binds to and neutralizes all anti-apoptotic Bcl-2-like proteins except Mcl-1 and A1. ABT-737 did not compromise GM-CSF-mediated survival when added to the culture either simultaneously (data not shown) or 4 h after stimulation start (allowing A1 and Mcl-1 induction before ABT-action) (Supplementary Figure S1E), indicating that GM-CSF-enhanced survival is independent of Bcl-2, Bcl-x_L_ or Bcl-2-like protein 2 (Bcl-w), but most likely entirely dependent on Mcl-1 and A1. In contrast, LPS-mediated survival was sensitive to ABT-737, with transient partial resistance at earlier stages, but almost no protection retained after 72 h (Supplementary Figure S1F). These findings correlated well with sustained upregulation of A1 and Mcl-1 in GM-CSF-treated cells, but transient induction by LPS.

### Regulation of A1 expression through kinase signaling pathways

**Figure 2:**
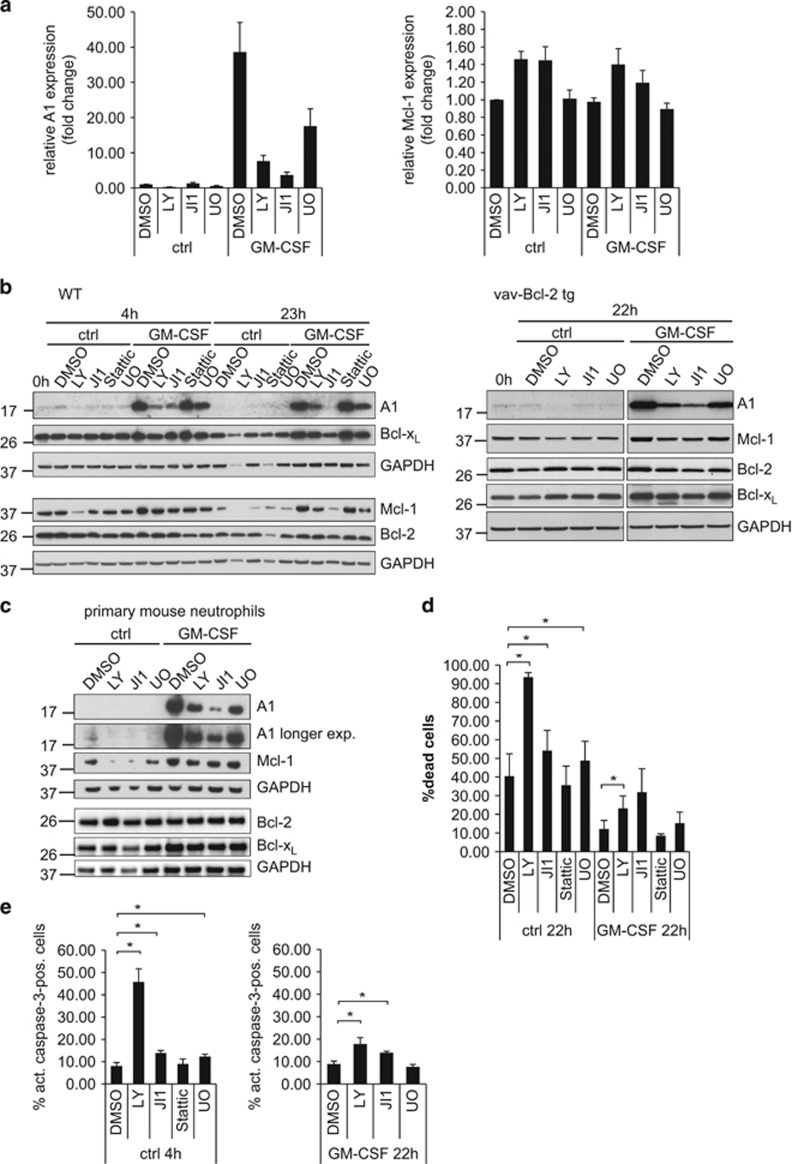
Regulation of A1 and Mcl-1 mRNA and protein levels by various signaling pathways in differentiated neutrophils and correlation with cell survival. (**a**) Relative A1 or Mcl-1 mRNA expression levels upon treatment with the kinase inhibitors. Day 4 differentiated Hoxb8 neutrophils were treated with LY294002 (LY, 20 *μ*M), JI1 (1 *μ*M) or U0126 (U0, 10 *μ*M) or solvent control (DMSO, 0.2%) for 4 h in the presence or absence of GM-CSF (1% B16-GM-CSF supernatant). After harvest, total RNA was isolated, reverse transcribed and real-time qRT-PCR was performed on a Light Cycler 2.0 instrument (Roche). Left, A1 mRNA, right, Mcl-1 mRNA expression as fold change normalized to *β*-actin are shown. Data represent mean/S.E.M. of three independent experiments. (**b**) A1 and Mcl-1 protein expression levels in day 4 differentiated wt (left) or vav-Bcl-2 transgenic (right) neutrophils upon treatment with kinase inhibitors as indicated in (**a**) for 4 or 23 h in the presence or absence of GM-CSF. Cells were lysed by resuspending and boiling cell pellets in Laemmli buffer. Equivalents of 1 × 10^6^ cells were separated by SDS-PAGE, transferred onto nitrocellulose membrane and subjected to immunoblotting against A1, Mcl-1, Bcl-2 (mouse-specific) and Bcl-x_L_. GAPDH served as a loading control. Data are representative of a total of 3–4 (WT cells) or two independent experiments (vav-Bcl-2 cells). (**c**) A1 and Mcl-1 protein levels in primary mature mouse neutrophils. Mouse neutrophils were sorted from bone marrow of C57BL/6 mice by positive selection and were treated with various inhibitors as indicated for 20 h. Cells were lysed by resuspending and boiling of harvested cell pellets in Laemmli buffer. Samples were separated by SDS-PAGE and transferred onto nitrocellulose membrane. Blots were probed for A1, Mcl-1, Bcl-2 (mouse-specific) and Bcl-x_L_. GAPDH served as a loading control. Data are representative of two independent experiments. (**d**) Influence of various kinase inhibitors on survival of differentiated neutrophils in the presence or absence of GM-CSF. Day 4 differentiated Hoxb8 neutrophils were were treated with inhibitors LY294002 (20 *μ*M), JI1 (1 *μ*M), Stattic (0.5 *μ*M) or UO126 (10 *μ*M) as indicated in the absence or presence of GM-CSF (1% B16-GM-CSF supernatant) for 20 h. Cell death was determined by staining with PI for loss of membrane integrity followed by flow cytometry analysis. Data show mean/S.E.M. of 3–5 independent experiments. *P≤0.05. (**e**) Apoptosis rate in differentiated neutrophils upon treatment with various kinase inhibitors. Day 4 differentiated Hoxb8 neutrophils were treated with various inhibitors as indicated above for 4 h (left) or 22 h (right). After harvest, cells were washed, permeabilised and subjected to intracellular staining against active caspase-3 as a marker of apoptosis. Data represent 3–4 independent experiments. *P≤0.05

The signaling pathways connecting Mcl-1 to pro-inflammatory receptors have been partially characterized, and signaling through PI3K, JAK/STAT, p38 and mitogen-activated protein kinase (MEK)/ERK pathways have been described.^29, 30^ The regulation of A1 during pro-inflammatory stimulation is much less understood. A significant decrease in A1 mRNA levels in unstimulated cells was observed upon PI3K inhibition, but not when JAK/STAT or MEK/ERK inhibitors were used. In GM-CSF-treated cells, the strong cytokine-induced upregulation of A1 mRNA expression was largely blocked by PI3K or JAK/STAT inhibition, and to a smaller extent with the MEK inhibitor U0126 (Figure 2a, left). In contrast, no reduction of Mcl-1 mRNA levels was observed with any of the inhibitors, neither in resting nor GM-CSF-treated neutrophils (Figure 2a, right).

We obtained similar results for A1 protein levels. The weak A1 protein expression seen in unstimulated neutrophils was further downregulated upon PI3K inhibition in wt and vav-Bcl-2 neutrophils (Figure 2b), correlating with mRNA levels. In GM-CSF-stimulated cells, the strong A1 protein induction was almost blocked by PI3K inhibitors or JAK inhibitor I (JI1), with JAK inhibition having the strongest effect. The good correlation with mRNA levels indicates mainly transcriptional regulation of A1. The STAT3 inhibitor Stattic had no effect on A1 protein or mRNA expression (Figure 2b and data not shown). Although on a smaller scale, similar signals as for A1 drove Mcl-1 protein levels during GM-CSF stimulation (Figure 2b). Mcl-1 protein expression was reduced by PI3K inhibition in unstimulated cells and by PI3K or JAK/STAT inhibition in GM-CSF-stimulated cells, as described earlier.^29, 30, 31^ In contrast to previous reports,^29^ no correlation between Mcl-1-mRNA and protein levels under pro-inflammatory stimulation and/or kinase inhibition was observed (Figures 2a and b). Regarding the anti-apoptotic proteins Bcl-2 and Bcl-x_L_, minor changes in protein levels were observed (Figures 2b and c). Similar results were obtained in primary mouse neutrophils. JAK inhibition during GM-CSF treatment caused the strongest reduction of A1 protein (Figure 2c), whereas effects of PI3K and JAK/STAT inhibition on Mcl-1 expression were less prominent.

Spontaneous neutrophil apoptosis was inversely correlated with both A1 and Mcl-1 expression. Inhibitor treatment in resting cells caused additional apoptosis, with the strongest effect by PI3K inhibition (Figures 2d and e), and moderate increase by JAK inhibition. In GM-CSF-stimulated cells, the pro-survival effect of GM-CSF was most strongly counter-regulated by JAK/STAT or PI3K inhibition, whereas MEK/ERK inhibition had minor effects (Figures 2d and e). Survival of GM-CSF-stimulated cells correlated best with A1 expression.

### A1 expression during differentiation of Hoxb8 neutrophils

**Figure 3:**
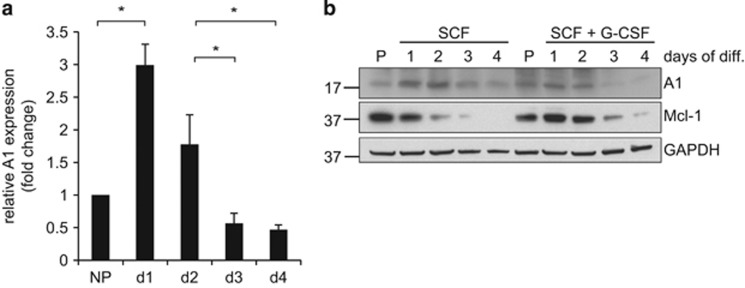
mRNA and protein expression levels of A1 in differentiating neutrophils. (**a**) relative mRNA expression levels of A1 during differentiation. Wt Hoxb8 progenitor cells were induced to undergo differentiation by estrogen withdrawal and monitored daily up to day 4. Cells were harvested at the indicated time points, followed by extraction of total RNA and reverse transcription into cDNA. Quantitative real-time RT-PCR was performed on a Light Cycler 2 instrument. Shown are A1 mRNA expression levels relative to *β*-actin expression normalized to progenitors (day 0). Data represent mean/S.E.M. of a total of 3–4 independent experiments. *P≤0.05. (**b**) Protein expression levels during differentiation of Hoxb8 neutrophils. Progenitor cells were induced to undergo differentiation by estrogen withdrawal either in the presence of SCF alone or with additional stimulation with G-CSF. Cells were harvested at the indicated time points and extracted by lysis and boiling in Laemmli buffer. Whole-cell lysates were separated by SDS-PAGE, transferred onto nitrocellulose membrane and probed for A1 and Mcl-1. GAPDH served as a loading control. Shown is one representative experiment out of three (SCF alone) or two (SCF+G-CSF)

We next assessed A1 levels during differentiation of neutrophils from progenitors. Differentiation was accompanied by the typical changes in cell size, cellular and nuclear morphology and surface marker expression as previously described^21^ and shown in Supplementary Figure S2. A1 mRNA was induced early during differentiation (days 1 and 2; Figure 3a). The same time course was seen at the protein level where A1 expression peaked at days 1 and 2. Additional G-CSF treatment during differentiation led to accelerated maturation (MacDonald *et al.*^32^; data not shown) and slightly faster decrease in A1 protein expression at later differentiation stages, but concomitantly to earlier onset of apoptosis (data not shown). Mcl-1 levels declined during differentiation as shown earlier.^21^

### Effect of pro-inflammatory stimulation on A1 and Mcl-1 protein levels in neutrophil progenitors and correlation with survival

**Figure 4:**
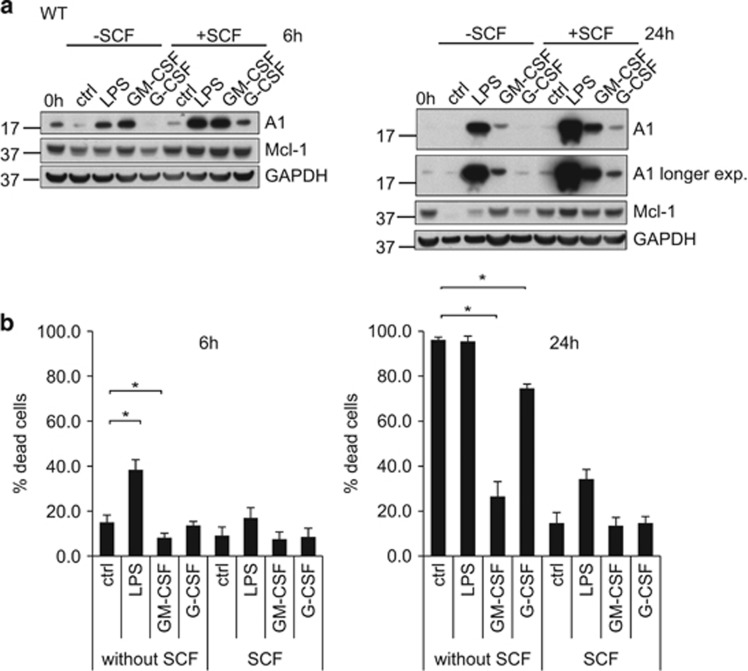
Effect of pro-inflammatory stimulation on protein expression levels in neutrophil progenitors and correlation with survival. (**a**) Protein expression levels in neutrophil progenitors. Wt progenitors were stimulated for 6 h with either LPS, GM-CSF or G-CSF or left untreated. In some samples, SCF was withdrawn. Cells were harvested at the time points indicated and extracted by direct lysis and boiling in Laemmli buffer. Whole-cell lysates (equivalents of 1 × 10^6^ cells per lane) were separated by SDS-PAGE, transferred onto a nitrocellulose membrane and probed for A1 and Mcl-1. GAPDH served as a loading control. Shown is one representative experiment out of three (GM-CSF) or two (LPS, G-CSF) independent experiments. (**b**) Impact of pro-inflammatory stimulation on survival of progenitors. Wt neutrophil progenitors were stimulated as indicated in **a**. Cells were harvested after 6 h (left panel) or 24 h (right panel) and subjected to propidium iodide staining followed by flow cytometry. Data comprise mean/S.E.M. of three independent experiments. *P≤0.05

By morphology and surface markers, Hoxb8 neutrophil progenitors correspond to an early promyelocyte stage. Progenitor stimulation with GM-CSF or LPS strongly induced A1 expression, whereas only slight induction was seen with G-CSF (Figure 4a). Analysis of vav-Bcl-2 progenitors gave similar results (data not shown). The regulation of Mcl-1 by these cytokines was very small (Figure 4a).

When stem cell factor (SCF) was withdrawn, most progenitors died within 24 h (Figure 4b). Surprisingly, and in contrast to differentiated neutrophils, LPS had a pro-apoptotic effect in progenitors. SCF withdrawal-induced cell death could partially be blocked by GM-CSF, but not by LPS and only little by G-CSF (Figure 4b).

**Figure 5:**
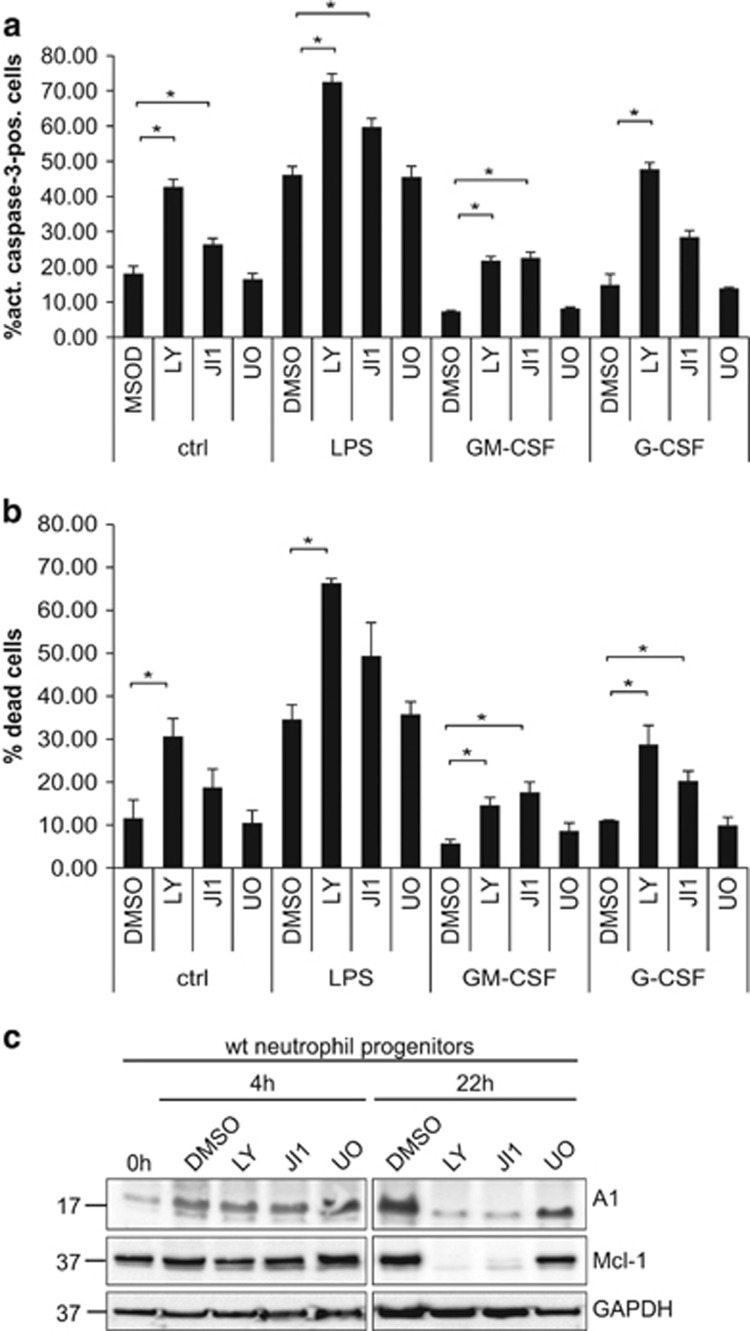
Effect of kinase inhibitors on survival of resting neutrophil progenitors and upon pro-inflammatory stimulation. (**a**) Wt progenitor cells were SCF withdrawn and treated for 6 h with various kinase inhibitors (LY294002 (20 *μ*M), JI1 (1 *μ*M), U0126 (10 *μ*M)) as indicated in the presence or absence of LPS, GM-CSF or G-CSF. Cells were harvested and analyzed for apoptosis by intracellular staining for active caspase-3 followed by flow cytometry analysis. Data represent mean/S.E.M. of three independent experiments. *P≤0.05. (**b**) Wt progenitor cells were treated as described in **a** for 6 h. Cells were harvested, stained for loss of membrane integrity by propidium iodide and analyzed by flow cytometry. Data represent mean/S.E.M. of four independent experiments. *P≤0.05. (**c**) Wt progenitors were treated with the kinase inhibitors LY294002 (20 *μ*M), JI1 (1 *μ*M), U0126 (10 *μ*M) or solvent control (DMSO, 0.2%) as indicated for 4 or 22 h. After harvest, cells were lysed and heated in Tricine sample buffer, and equivalents of 0.6 × 10^6^ cells were subjected to SDS-PAGE on 16% Tricine gels, transferred onto a nitrocellulose membrane and probed for A1 and Mcl-1. GAPDH served as loading control. Shown is one out of three independent experiments

In the absence of further stimulation, PI3K inhibition had a strong pro-apoptotic effect, and JAK inhibition also enhanced cell death. The pro-survival effect of GM-CSF and G-CSF on progenitors was reduced when PI3K or JAK were inhibited, whereas LPS-induced cell death was considerably enhanced (Figures 5a and b). LY and JI1 reduced Mcl-1 expression, which also correlated with cell death. (Figure 5c). The already very low expression levels of A1 were not consistently altered early after inhibitor treatment, but apparently decreased under conditions of PI3K or JAK inhibition at later time points (Figure 5c).

### ShRNA-mediated KD of A1 and Mcl-1 in neutrophil progenitors and differentiating cells

**Figure 6:**
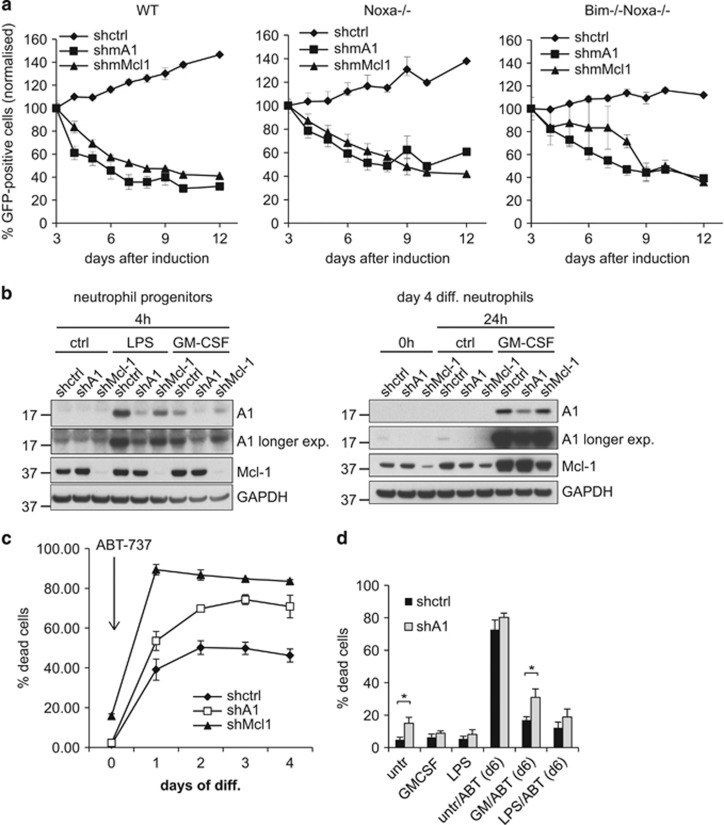
KD of A1 by shRNA sensitizes progenitors, early differentiating cells and mature neutrophils upon pro-inflammatory stimulation to apoptosis. (**a**) shRNA-mediated KD in progenitors. Wt, Noxa−/− or Bim−/−Noxa−/− Hoxb8 neutrophil progenitors were lentivirally transduced with shRNA directed against A1, Mcl-1 or an irrelevant non-mammalian control sequence (shctrl). The lentiviral construct results in constitutive expression of the shRNA and includes GFP as a marker. After transduction, polyclonal cell populations were followed over time for GFP positivity after transduction. Shown is percentage of GFP-positive cells of total living cells at the time points indicated. Data were normalized to day 3 after transduction (100%) to account for variation in transduction efficiencies. Data represent mean/S.E.M. of a total of three independent experiments, respectively. (**b**) shA1 and shMcl-1 KD efficiency on the protein level in vav-Bcl-2-tg neutrophil progenitors (left) and differentiated neutrophils (right). Cells had been sorted for >90% of GFP positivity by flow cytometry. Cells were stimulated as indicated, harvested at the indicated time points and subjected to immunoblot analysis for A1 and Mcl-1. GAPDH served as a loading control. Data are representative of three independent experiments. (**c**) ‘Activation' of KD during differentiation. Vav-Bcl-2-tg neutrophil progenitors constitutively expressing either shA1, shMcl-1 or a ctrl shRNA were induced to undergo differentiation by estrogen withdrawal and were at the same time treated with ABT-737 (1 *μ*M) starting at day 0 of differentiation. Cell death was determined at day 3 of differentiation by staining for loss of cell membrane integrity by propidium iodide followed by flow cytometry analysis. Data represent mean/S.E.M. of three independent experiments. (**d**) Effect of A1 KD in mature neutrophils upon pro-inflammatory stimulation. Differentiated vav-Bcl-2-tg Hoxb8 neutrophils stably expressing shRNA against A1 or shctrl were treated with GM-CSF or LPS, respectively, at day 4 of differentiation. Cells were in addition treated with ABT-737 (1.5 *μ*M) or solvent control at day 6 and were analyzed for cell death by propidium iodide staining and flow cytometry 24 h later. Shown are mean/S.E.M. of four (LPS) or five (GM-CSF) independent experiments performed. *P<0.05 (Student's *t*-test)

In order to test the relevance of A1 at different stages of neutrophil development and activation, we targeted all isoforms in the Hoxb8 system using RNAi. First, shRNA-mediated KD of A1 and Mcl-1 was done by lentiviral transduction using enhanced green fluorescent protein (EGFP) as marker. Wt neutrophil progenitors were monitored for EGFP expression after transduction. The proportion of EGFP-expressing cells decreased over time both in shA1 and shMcl-1-transduced populations at similar rates (Figure 6a), whereas control shRNA (shctrl) cells showed even a slight relative increase over time. The Bcl-2 homology domain (BH3)-only proteins Bim (Bcl-2 interacting mediator of cell death) and Noxa (phorbol-12-myristate-13-acetate-induced protein 1) are the most important pro-apoptotic regulators of neutrophil apoptosis.^21, 33^ To test whether loss of these BH3-only proteins could rescue cells from effects of A1 or Mcl-1 loss, Noxa single- or Bim/Noxa double-deficient cells were analyzed. Neither loss of Noxa alone nor combined loss of Bim and Noxa was able to prevent loss of EGFP-expressing cells (Figure 6a). This suggests that the loss of either protein acts downstream of BH3-only proteins and implicates that A1, as it has been suggested for Mcl-1,^34^ may directly bind to and inhibit the downstream effectors Bax (Bcl-2-associated X protein) and Bak (Bcl-2 homologous antagonist/killer; Figure 6a).

We also attempted to establish a Tet-inducible KD system based on the TetRKR repressor.^35^ Induction of either A1 or Mcl-1 KD led to decrease of GFP-positive transduced progenitors (Supplementary Figure S4), similar to constitutive expression, but we were not able to maintain Tet-inducible cell lines for longer time periods, most likely due to toxicity of the Tet repressor.

We therefore established an inducibly ‘activatable' KD system by constitutive expression in Bcl-2-overexpressing progenitors. In these cells, shRNA KD should have no impact on survival (as any cell death induced by A1 loss would be inhibited by Bcl-2). As soon as Bcl-2 function is inhibited by treatment with ABT-737, Bcl-2-family-mediated survival of the cells will depend exclusively on the anti-apoptotic proteins not targeted by ABT-737,^28^ that is, Mcl-1 and A1. ShA1 or shMcl-1-expressing progenitor lines with efficient and stable KD could be generated (Figure 6b). When treated with ABT-737, these cells showed dose-dependent induction of cell death (Supplementary Figure S5). Death was slightly enhanced in A1-KD cells and strongly enhanced in Mcl-1-KD cells compared with shctrl cells. This confirmed the crucial role of Mcl-1 in progenitor survival, but also indicated a role of A1 for survival of progenitors. KD was also detectable in differentiated cells, although somewhat less efficient, indicating possible counterselection over time (Figure 6b). When the KD was 'activated' by ABT-737 at differentiation start, significantly enhanced cell death was observed in A1-KD cells (Figure 6c). The strongest effect was seen, as expected, in Mcl-1-KD cells (Figure 6c). The impact of A1 KD on survival of differentiating neutrophils thus correlates with A1 mRNA and protein levels, peaking at days 1 and 2 of differentiation. Data indicate that besides Mcl-1, A1 also is an important regulator of survival of differentiating neutrophils.

To further address the relevance of A1 for enhanced survival of mature neutrophils upon pro-inflammatory stimulation, differentiated vav-Bcl-2-tg A1-KD cells were pre-stimulated with GM-CSF or LPS and then treated with ABT-737. ABT-737 treatment of otherwise untreated neutrophils led to strong cell death, which was comparable in shctrl and A1-KD cells (Figure 6d). Preceding stimulation with GM-CSF strongly protected shctrl cells against ABT-737-induced killing. When A1 was knocked down, this protective effect was partially, but significantly, reduced (Figure 6d). Under LPS stimulation, ABT-737-treated A1-KD neutrophils showed a slight, nonsignificant decrease in survival. Mcl-1-KD, as expected, led to a strong reduction of the pro-survival effect of pro-inflammatory stimulation, although to a lesser extent in case of LPS-stimulated compared with GM-CSF-stimulated survival (Supplementary Figure S6). Results obtained in the shRNA-KD system thus indicate that A1 is important for survival in the neutrophil lineage especially early in differentiation, but also contributes to the strong pro-survival effect of pro-inflammatory stimulation.

### Regulation of A1 in human neutrophils

**Figure 7:**
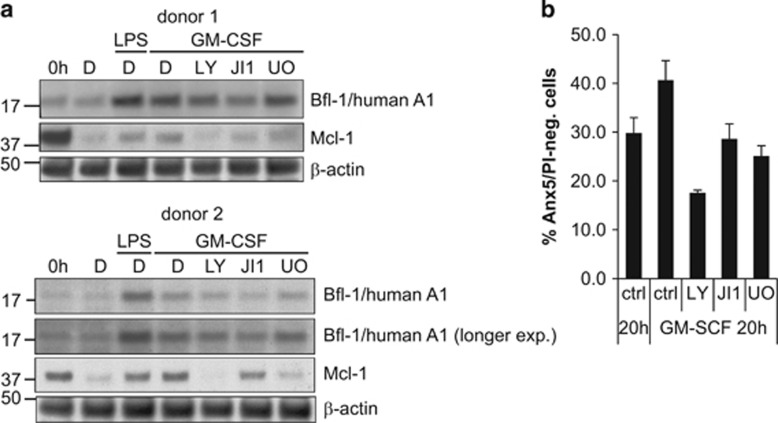
A1 protein expression in human primary neutrophils upon pro-inflammatory stimulation and inhibitor treatment. (**a**) Immunoblot analysis of human peripheral blood-derived primary neutrophils. Human neutrophils were isolated from peripheral blood of two different healthy donors by Histopaque/Percoll density centrifugation and were stimulated for 20 h with human recombinant GM-CSF (10 ng/ml) or LPS (1 *μ*g/ml) where indicated. Cells were additionally treated with LY (20 *μ*M), JI1 (1 *μ*M) or U0126 (10 *μ*M) or solvent control (0.5% DMSO). Proteins were extracted by lysis and boiling in Laemmli buffer. Whole-cell lysates (equivalents 1 × 10^6^ cells per lane) were separated by SDS-PAGE, transferred onto a nitrocellulose membrane and probed for Bfl-1/A1 and Mcl-1. *β*-Actin served as a loading control. Shown are blots derived from cells of two healthy individuals. (**b**) Survival of human primary neutrophils during pro-inflammatory stimulation in the presence or absence of various kinase inhibitors. Cells isolated from human healthy donors as described in **a** were stimulated with human recombinant GM-CSF or not in the presence of kinase inhibitors or solvent control as indicated above for 20 h. Apoptosis was analyzed by Annexin V/PI staining followed by flow cytometry. Data show survival as determined by the percentage of Annexin V/PI-negative cells. Bars represent mean/S.E.M. of two experiments with cells from two donors each (*n*=4 in total)

Primary human neutrophils isolated from peripheral blood were included in the analysis. A1 protein could be detected by immunoblotting. The protein was strongly induced by pro-inflammatory stimuli (Figure 7). Simultaneous GM-CSF stimulation and treatment with either LY, JI1 or U0126 led to decreased A1 expression with the most consistent effect when the JAK/STAT pathway was blocked. Mcl-1 protein was also strongly regulated, but here PI3K inhibition caused the strongest reduction of Mcl-1 expression. The pro-survival effect of GM-CSF stimulation in human neutrophils was blocked when any of the PI3K, JAK/STAT or MEK/ERK pathways was inhibited (see Figure 7b). The results strongly suggest that A1 is an important anti-apoptotic factor, which cooperates with Mcl-1 in regulation of neutrophil survival and homeostasis. A1 may thus be a potentially interesting target for anti-inflammatory therapy in neutrophil-dependent inflammatory diseases.

## Discussion

Here we analyzed endogenous A1 expression and its regulation at the protein level in neutrophils. We identified a major role of PI3K and JAK/STAT signaling pathways for A1 regulation and show that A1 is important for survival of differentiating and mature neutrophils. The results confirm the importance of Mcl-1 for survival of progenitors committed to the neutrophil lineage^9^ but extend this role to all stages of differentiation including mature neutrophils. Mcl-1 conveys the strongest pro-survival effect at all stages of neutrophil development. A1, however, considerably contributes to modulation of neutrophil lifespan.

The upregulation of A1, observed early during differentiation, correlated well with compromised survival of A1 KD cells. This suggests an important function for A1 in maintenance of cell survival during neutrophil differentiation.

Strikingly, A1 upregulation by LPS and GM-CSF was much stronger than Mcl-1 regulation, and A1 KD sensitized to apoptosis in the presence of LPS and GM-CSF, linking A1 to enhanced neutrophil survival in response to microbial and other pro-inflammatory stimuli. Surprisingly and contrary to the effect in differentiated neutrophils, LPS did not protect, but even caused cell death in progenitors. This finding may have clinical impact as cytotoxic effects on progenitors could contribute to development of leukopenia observed in some sepsis patients.^36^

In contrast to GM-CSF, G-CSF failed to upregulate of A1 in primary neutrophils and caused rather weak A1 induction in progenitors. G-CSF and GM-CSF are clinically relevant therapeutics for treatment of acquired and congenital neutropenia and for chemotherapy- or irradiation-induced neutropenia, respectively.^37^ A1 is likely involved in the therapeutic effect of GM-CSF on progenitors and differentiated cells, whereas the G-CSF-mediated pro-survival effect probably is rather Mcl-1 dependent then A1 dependent.

Both Mcl-1 and A1 protein levels correlated with neutrophil survival. Regulation of A1 and Mcl-1 was, however, substantially different, which suggests complementary, only partially redundant functions. A striking difference between A1 and Mcl-1 regulation is the strong transcriptional control of A1 in contrast to primarily posttranscriptional regulation of Mcl-1. Whereas the mRNAs of all murine and human A1 isoforms have very short 3′UTRs of <200 bp, Mcl-1 mRNA contains a 3′UTR of >2000 bp, with presumably more regulatory elements. This is consistent with more pronounced regulation of Mcl-1 at the posttranscriptional level, for example, by microRNAs.

Both Mcl-1 and A1 have short half-lifes. Several regulators of Mcl-1 at the level of protein turnover have been identified.^38^ A1 is also reported to undergo ubiquitination followed by strong proteasomal degradation.^39, 40^ Both proteins are thus turned off rapidly when transcription or translation is blocked, which may explain the fast onset of apoptosis in neutrophils deprived of survival factors.

Several STATs are downstream targets in G-CSF and GM-CSF signaling. STAT1, 3 and 5 activation have been reported for G-CSF,^41^ and GM-CSF has been shown to signal mainly through STAT5A/B,^42^ but also STAT1 and 3.^43^ Our results argue against involvement of STAT3 in A1 regulation in neutrophils. The differential regulation of A1 by GM-CSF *versus* G-CSF may be explained by preferential signaling via STAT3 by G-CSF and mainly STAT5-mediated signaling by GM-CSF, resulting in more pronounced Mcl-1, but only weak A1 induction by G-CSF, in contrast to strong GM-CSF-mediated A1 upregulation.

Regulation of A1 expression by STATs has previously been described in other cell types.^44, 45, 46^ However, *in silico* analyses of the A1 promoter did not reveal STAT binding sites (data not shown). Direct targeting of the A1 promoter by STATs cannot be excluded, but indirect regulation of A1 via JAK/STAT is also possible. Candidates for such indirect regulation include c/EBPs and Pu.1 transcription factors, which are reported to be regulated by JAK/STAT and to have A1 as transcriptional target in certain situations.^47, 48, 49^ C/EBPs and Pu.1 thus are potential downstream targets for A1 regulation of in the neutrophil lineage.

Targeting of Mcl-1 is experimentally possible,^50, 51^ and a potential A1 inhibitor has been described.^52^ Although not suitable for therapeutic use, this agent may be a potential lead for further therapeutic development.

PI3K or ERK inhibitors have been suggested as anti-inflammatory agents acting by blocking neutrophil recruitment or promoting resolution of inflammation.^53, 54^ JAK inhibitors already have a role in combatting myeloproliferative disease and immunological disorders.^55, 56, 57^ Part of the therapeutic effect of these inhibitors is possibly due to inhibition of A1 and/or Mcl-1 activity.

JAK inhibitors as well as specific A1 inhibitors may become part of future therapeutic strategies against diseases related to neutrophilic inflammation. In contrast, inhibition of apoptosis by restoring compromised A1 and Mcl-1 activity may be useful in situations of disturbed neutrophil survival and function.

## Materials and Methods

### Cell lines and cell culture

Hoxb8 neutrophil progenitors were derived from bone marrow of C57BL/6 wt or vav-Bcl-2-tg mice^24^ expressing Bcl-2 throughout the hematopoietic lineage. Polyclonal neutrophil progenitor cell lines were established by retroviral transduction of Hoxb8 and selection in the presence of SCF.^20^ Progenitor cells were cultured in Opti-MEM medium (Life Technologies, Carlsbad, CA, USA) supplemented with 10% FCS, 30 mM *β*-mercaptoethanol, 1% supernatant from SCF-producing CHO cells (kindly provided by Hans Häcker) and 1 *μ*M *β*-estradiol (Sigma-Aldrich, Munich, Germany). Differentiation was induced by estrogen removal and culture in medium containing 1% SCF supernatant. Murine GM-SCF was either used as 1% cell culture supernatant of GM-CSF-producing B16 cells corresponding to a final concentration of approximately 10 ng/ml according to Wang *et al.* ^20^ (kindly provided by Hans Häcker), or as commercially available recombinant murine GM-CSF at a concentration of 10 ng/ml (Peprotech, Hamburg, Germany). Both GM-CSF preparations led to comparable results. Human recombinant GM-CSF (Peprotech), murine recombinant G-CSF (Peprotech), LPS (*E. coli* O55:B5, Sigma-Aldrich cat no. L2637, Munich, Germany) or the inhibitors LY94002 (20 *μ*M, Sigma-Aldrich) Jak inhibitor 1 (JI1, 1 *μ*M), Stattic (0.5 *μ*M) and U0126 (10 *μ*M, all from Calbiochem, San Diego, CA, USA) were used as indicated.

### Isolation of primary mouse or human neutrophils

Primary mouse neutrophils were sorted from bone marrow of C57BL/6 mice by positive selection using the Miltenyi MACS purification kit (Miltenyi Biotech, Bergisch Gladbach, Germany). Following harvest of bone marrow cells and red blood cell lysis, neutrophils were labeled with FITC-coupled anti-Gr-1 antibody (BD Biosciences, San Jose, CA, USA), followed by anti-FITC antibody coupled to magnetic beads and isolated by passage over MACS columns (Miltenyi Biotech). Gr-1-positive cells were cultured in complete RPMI medium and analyzed as indicated.

Human neutrophils were isolated from peripheral blood from healthy adult donors by discontinuous density gradient centrifugation essentially as described.^58^

### Monitoring of cell differentiation by Giemsa staining and cell surface marker expression

Staining for cellular and nuclear morphology was performed on cytospins from cultures of progenitors or differentiated neutrophils by incubating with Giemsa solution (Merck, Darmstadt, Germany) after methanol fixation. Analysis by brightfield microscopy was performed using a Keyence BZ9000 microscope at a magnification of x40 (Keyence, Neu-Isenburg, Germany).

Expression of cell surface markers was measured by staining cells with anti-Gr-1-FITC (BD Biosciences, San Jose, CA, USA), anti-CD11b-APC (eBioscience, San Diego, CA, USA), anti-c-kit-APC (eBioscience) or anti-CXCR2-APC (Biolegend, San Diego, CA, USA) followed by flow cytometry analysis on a FACS Calibur (BD Biosciences, Heidelberg, Germany).

### Lentiviral transduction for generation of shRNA KD cells

A lentiviral system^35^ was used to target A1 and Mcl-1 by shRNA (targeting sequences: A1: 5′-GAGTTGCTTTCTCCGTTCA-3′, Mcl-1: 5′-GGGACTGGCTTGTCAAACA-3′, control: 5′-GTATCATCTCTTGAATGAT-3′). ShRNA sequences were cloned into pENTR-THT and transferred via Gateway LR recombinase reaction (Invitrogen) into the lentiviral destination vector pHR-dest-SFFV-eGFP.^35^ ShRNA expression vectors were transfected into HEK 293FT cells (Invitrogen, Carlsbad, CA, USA), together with packaging plasmids psPAX2 and pMD2.G using Fugene HD (Roche, Mannheim, Germany). Lentiviral supernatants were harvested on days 2 or 3, filtered, and transduced at a cell density of 0.5–1 × 10^5^/ml in the presence of 5 *μ*g/ml polybrene. GFP-positive cells were sorted by flow cytometry and populations with >90% GFP positivity were used where indicated.

### Apoptosis and cell death assays

For staining of active caspase-3, cells were washed with PBS, fixed in 2% formaldehyde and permeabilised with 0.5% saponin (Sigma-Aldrich). Cells were incubated with anti-active caspase-3 (BD Pharmingen, Heidelberg, Germany) in PBS/0.5% BSA/0.5% saponin for 30 min, stained with anti-rabbit-Alexa-Fluor647 (Dianova GmbH, Hamburg, Germany) for 30 min and analyzed by flow cytometry.

In most experiments, cell death was assessed by propidium iodide staining for loss of cell membrane integrity. Cells were harvested in culture media, and PI was added at a final concentration of 1 *μ*g/ml before analysis by flow cytometry on a FACS Calibur. In some experiments, cell death by loss of cell membrane integrity was quantified using the LIVE/DEAD Fixable Far Red Dead Cell Stain (Molecular Probes, Thermo Fisher Scientific, Waltham, MA, USA) according to the manufacturer's instructions.

Annexin V-propidium iodide staining was done by washing cells with Annexin V-binding buffer (eBioscience) and staining with Annexin V-FITC (1 : 50; BD Pharmingen) 20 min at 4 °C. Propidium iodide (1 *μ*g/ml; Sigma-Aldrich) was added and cells were analyzed on a FACS Calibur (Becton Dickinson, Heidelberg, Germany).

### RNA extraction and qRT-PCR

Total RNA was extracted and transcribed into cDNA with commercial kits (Roche). Samples were analyzed by quantitative RT-PCR on a Light Cycler 2 using the Lightcycler Taqman Master kit and the universal probe library (Roche) (Mcl-1 and *β*-actin). For quantification of A1, Taqman gene expression assays (Applied Biosystems, Foster City, CA, USA) were used. Relative expression of the gene of interest was normalized to *β*-actin expression.

### Immunoblot analysis

Cells were harvested with accutase treatment when adequate, washed with PBS, lysed and heated at 95 °C for 5 min (Laemmli buffer), 70 °C for 10 min (Bolt sample buffer) or 85 °C for 2 min (Tricine sample buffer). Extracts were separated by SDS-PAGE either on 12.5% gels (EZ-run, Fisher Scientific, Schwerte, Germany), 4–12% Bolt gels (Life Technologies) or 16% Tricine gels (Life Technologies), and proteins were transferred onto nitrocellulose membranes (0.2 *μ*m). Membranes were probed with anti-mouse A1 (rat monoclonal, kindly provided by Marco Herold, WEHI, Melbourne, Australia), anti-human A1 (rabbit polyclonal, kindly provided by Jannie Borst), anti-mouse Mcl-1 (Rockland, Limerick, PA, USA, #600-401-394), anti-human Mcl-1 (BD, Biosciences, Heidelberg, Germany, #559027), anti-mouse Bcl-2 (BD, clone 3F11), anti-Bcl-x_L_ (NEB, Frankfurt, Germany, clone 54H6) or anti-GAPDH (Millipore, Darmstadt, Germany, clone 6C5) or anti-*β*-actin (Sigma, clone AC-15) antibodies. Proteins were visualized using peroxidase-conjugated anti-rabbit (Sigma), anti-mouse (Dianova), anti-hamster (Dianova) or anti-rat (NEB) antibodies by enhanced chemoluminescence detection (ECL Prime, GE Healthcare, Dornstadt, Germany; SuperSignal West Femto Substrate, Pierce, Fisher Scientific, Schwerte, Germany).

## Abbreviations

A1/Bfl-1: Bcl-2-related protein A1
Bak: Bcl-2 homologous antagonist/killer
Bax: Bcl-2-associated X protein
Bcl-2: B-cell lymphoma protein 2
Bcl-x_L_: Bcl-2-like protein 1 isoform 1
Bcl-w: Bcl-2-like protein 2
BH3: Bcl-2 homology domain
Bim: Bcl-2 interacting mediator of cell death
EGFP: enhanced green fluorescent protein
ERK: extracellular signal-regulated kinase
G-CSF: granulocyte colony-stimulating factor
GM-CSF: granulocyte-macrophage colony-stimulating factor
JAK: Janus kinase
JI1: JAK inhibitor 1
KD: knockdown
LPS: lipopolysaccharide
Mcl-1: induced myeloid leukemia cell differentiation protein
MEK: mitogen-activated protein kinase
Noxa: phorbol-12-myristate-13-acetate-induced protein 1
PI3K: phosphoinositide-3 kinase
SCF: stem cell factor
STAT: signal transducer and activator of transcription
wt: wild type
