# Supplementary material for: The anti-apoptotic Bcl-2 family protein A1/Bfl-1 regulates neutrophil survival and homeostasis and is controlled via PI3K and JAK/STAT signaling
Source: Cell Death Dis. 2016 Feb 18;7(2):e2103–. doi: 10.1038/cddis.2016.23 (PMC5399193; doi:10.1038/cddis.2016.23)
Supplement: Supplementary Figures [file cddis201623x1.pdf]

Supplementary figure legends revised

Fig. S1: B16-derived GM-CSF supernatant and recombinant murine GM-CSF exhibit comparable effects

A, Comparison of A1 and Mcl-1 protein expression levels in day 4 differentiated neutrophils stimulated with GM-CSF supernatant derived from B16 cells or with recombinant murine GM-CSF analyzed by immunoblotting. WT (upper panel) or vav-Bcl-2 transgenic (lower panel) differentiated Hoxb8 neutrophils were either treated with 1% GM-CSF supernatant derived from B16 cells (GM-CSF SN) or with recombinant murine GM-CSF at a concentration of 10 ng/ml (rm GM-CSF), respectively, or left untreated, for the indicated time periods. Cells were lysed by boiling in Laemmli buffer, and equivalents of $1x10^6$ cells/ lane were separated by SDS-PAGE, transferred onto nitrocellulose membrane and subjected to immunoblot analysis for A1 and Mcl-1. GAPDH served as a loading control. Data are representative for 2 independent experiments, respectively.

B, Viability of differentiated wt or vav-Bcl-2 transgenic Hoxb8 neutrophils upon stimulation

Day 4 differentiated wt (left panel) or vav-Bcl-2 transgenic (right panel) Hoxb8 neutrophils were either stimulated with 1% GM-CSF supernatant (derived from B16 cells, GM-CSF SN), with recombinant murine GM-CSF (10 ng/ml, rm GM-CSF) or with LPS (Sigma, 1 µg/ml) or left untreated and were monitored for up to 72h. Cells were harvested at the time points indicated and stained for loss of cell membrane integrity using the LIVE/DEAD Fixable Far Red Dead Cell Stain (Molecular Probes) followed by flow cytometry analysis. Data represent mean/SEM of n=4-5 experiments for wt cells or one of two independent experiments showing similar results for vav-Bcl-2 transgenic cells.

C, Quantification of apoptosis by detection of active caspase-3 in unstimulated or stimulated wt or vav-Bcl-2 transgenic Hoxb8 neutrophils. Wt or vav-Bcl-2 transgenic Hoxb8 neutrophils differentiated for 4 days by estrogen withdrawal were treated with either 1% GM-CSF supernatant, 10 ng/ml recombinant murine GM-CSF or 1 µg/ml LPS or left untreated for 18 hours. After harvest, fixation and permeabilisation, cells were subjected to staining against active Caspase-3 followed by flow cytometry analysis.

D, Comparison of A1 and Mcl-1 protein expression levels in day 4 differentiated wt neutrophils upon treatment with kinase inhibitors as indicated for 4h in the presence of either 1% GM-CSF SN or

recombinant murine GM-CSF at a concentration of 10 ng/ml (rm GM-CSF). Cells were lysed by resuspending and boiling cell pellets in Laemmli buffer. Equivalents of $1x10^6$ cells were separated by SDS-PAGE, transferred onto nitrocellulose membrane and subjected to immunoblotting against A1 and Mcl-1. GAPDH served as a loading control.

E, GM-CSF stimulated cells remain viable despite ABT-737 treatment. Day 4 differentiated Hoxb8 neutrophils were stimulated with 1% GM-CSF SN or with recombinant murine GM-CSF at a concentration of 10ng/ml (rm GM-CSF) and were additionally treated with ABT-737 (1,5 µM) or solvent control (DMSO) 4 hours after start of stimulation. Cells were monitored for loss of cell membrane integrity using the LIVE/DEAD Fixable Far Red Dead Cell Stain (Molecular Probes) and flow cytometry analysis at the time points indicated for up to 72h. Data represent mean/SEM of 3 independent experiments.

F, LPS stimulated neutrophils are partially sensitive to ABT-737 treatment. Day 4 differentiated wt Hoxb8 neutrophils were stimulated LPS (1 µg/ml) or not and were additionally treated with ABT-737 (1,5 µM) or solvent control (DMSO 0,3%) 4 hours after start of stimulation. Cells were monitored for loss of cell membrane integrity using the LIVE/DEAD Fixable Far Red Dead Cell Stain (Molecular Probes). Samples were harvested at the time points indicated for up to 72h and were analysed by flow cytometry. Data represent mean/SEM of 3 independent experiments.

Fig. S2: Characterisation of Hoxb8 neutrophil differentiation by morphology and surface markers

A, wt or vav-Bcl-2 transgenic Hoxb8 cells were either induced to undergo differentiation by estrogen withdrawal or cultured in the progenitor state. Progenitors and day 4 differentiated neutrophils (top panels) as well as bone-marrow sorted primary murine granulocytes (bottom panel) were monitored for cellular and nuclear morphology by Giemsa staining of cytospins. Brightfield images were taken with a Keyence BZ9000 microscope at a magnification of 40x (Scale bar = 20 µm). Data are representative of at least 3 experiments performed.

B, wt or vav-Bcl-2 transgenic Hoxb8 cells were induced to undergo differentiation by estrogen withdrawal as in A. Progenitors (red lines) and day 4 differentiated neutrophils (blue lines) were stained for surface expression of the markers CD11b, Gr-1, c-kit or CXCR2 and analysed by flow cytometry. Data are representative of at least 3 independent experiments performed.

Fig. S3: Time course of A1 protein induction by LPS stimulation and survival in primary murine bone marrow sorted neutrophils

A, Primary mouse neutrophils sorted from murine bone marrow were seeded at a density of 2,5 Mio cells/2,5ml and stimulated with LPS (1 µg/ml) for the time periods indicated or left untreated for 4 hours. Cells were harvested and directly lysed in Laemmli buffer. Equivalents of $6x10^5$ cells were separated by SDS-PAGE, transferred onto nitrocellulose membrane and subjected to immunoblotting against A1 and Mcl-1. GAPDH served as a loading control. Data are representative of 3 independent experiments performed with cells pooled from 2-3 mice per experiment, respectively.

B, Primary bone marrow-sorted neutrophils were stimulated with LPS or recombinant murine GM-CSF (10 ng/ml) or left untreated for 20h. Cell death of was monitored by propidium iodide staining for loss of cell membrane integrity followed by flow cytometry analysis. Data represent mean/SEM of a total of 4-6 mice analysed separately. *p<0,05 (students t-test).

Fig. S4: Time course of GFP-positivity after knockdown induction in doxycyline-inducible TRKR-shRNA neutrophil progenitors

Wt neutrophil progenitors expressing TRKR and conditional shRNA against A1 or Mcl-1 or control shRNA were induced by doxycycline (1µg /ml). Percentage of GFP-positive cells was monitored over the next 4 days by flow cytometry. Data are normalised to 100% at day 1 after induction to control for different transduction efficiencies and background cell death.

Fig. S5: “Activation” of A1- or Mcl-1-KD by ABT-737 in vav-Bcl-2-transgenic shRNA expressing neutrophil progenitors

Vav-Bcl-2-transgenic neutrophil progenitors constitutively expressing shRNA against A1, Mcl-1 or control shRNA were treated with various concentrations of ABT-737 in order to neutralize Bcl-2. Cell death (% of PI-negative cells) was measured after 5 hours by flow cytometry.

Fig. S6: Effect of Mcl-1 knockdown in mature neutrophils during proinflammatory stimulation

Day 4 differentiated vav-Bcl-2-tg Hoxb8 neutrophils stably expressing shRNA against Mcl-1 or control shRNA were treated with GM-CSF or LPS, respectively, at day 4 of differentiation. Cells were then treated with ABT-737 (1,5 µM) or solvent control at day 6 and were analysed for cell death 24

hours later by propidium iodide staining and flow cytometry. Shown are mean/SEM of 4-5 independent experiments performed.

Fig. S1

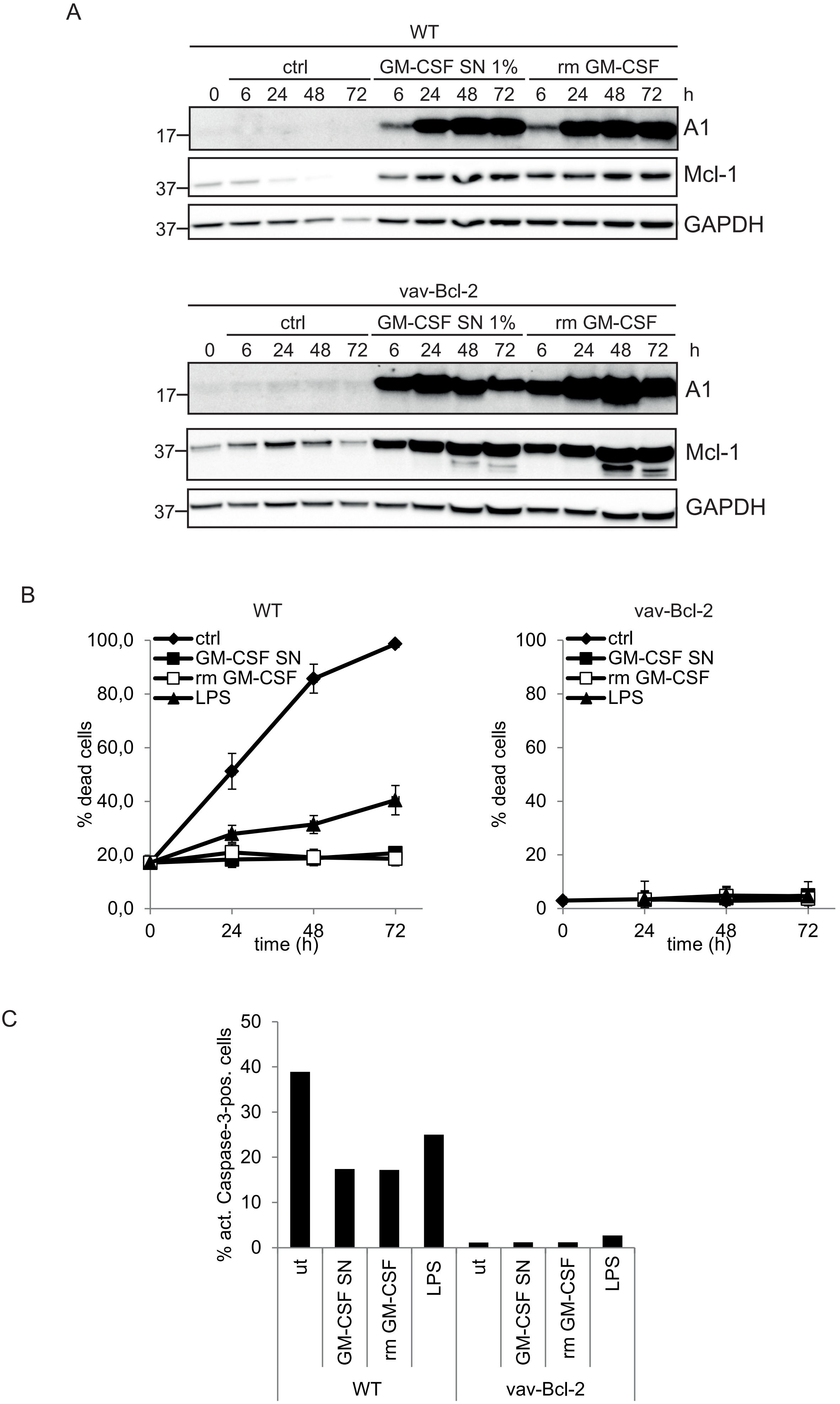
A
WT
ctrl
GM-CSF SN 1%
rm GM-CSF
0 6 24 48 72 6 24 48 72 6 24 48 72 h
17
A1
37
Mcl-1
37
GAPDH
vav-Bcl-2
ctrl
GM-CSF SN 1%
rm GM-CSF
0 6 24 48 72 6 24 48 72 6 24 48 72 h
17
A1
37
Mcl-1
37
GAPDH
B
WT
ctrl
GM-CSF SN
rm GM-CSF
LPS
% dead cells
0,0
20,0
40,0
60,0
80,0
100,0
0 24 48 72
time (h)
vav-Bcl-2
ctrl
GM-CSF SN
rm GM-CSF
LPS
% dead cells
0
20
40
60
80
100
0 24 48 72
time (h)
C
% act. Caspase-3-pos. cells
0
10
20
30
40
50
ut
GM-CSF SN
rm GM-CSF
LPS
ut
GM-CSF SN
rm GM-CSF
LPS
WT
vav-Bcl-2

Fig. S1

D

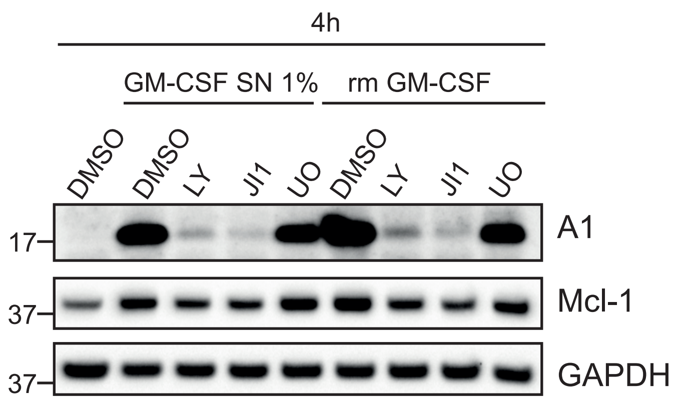
4h
GM-CSF SN 1%
rm GM-CSF
DMSO
DMSO
LY
JI1
UO
DMSO
LY
JI1
UO
17
37
37
A1
Mcl-1
GAPDH

E

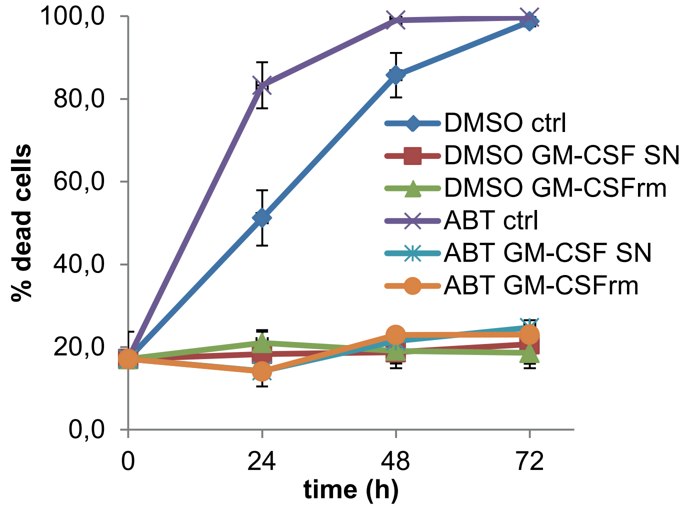
% dead cells
100,0
80,0
60,0
40,0
20,0
0,0
0
24
48
72
time (h)
DMSO ctrl
DMSO GM-CSF SN
DMSO GM-CSFrm
ABT ctrl
ABT GM-CSF SN
ABT GM-CSFrm

F

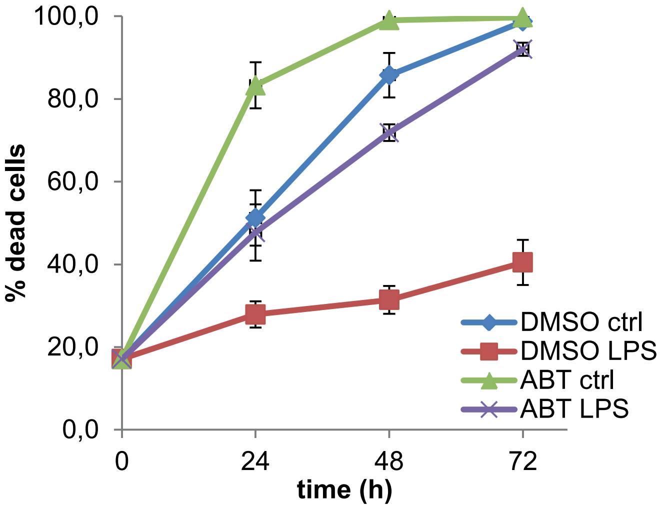
% dead cells
100,0
80,0
60,0
40,0
20,0
0,0
0
24
48
72
time (h)
DMSO ctrl
DMSO LPS
ABT ctrl
ABT LPS

Fig. S2

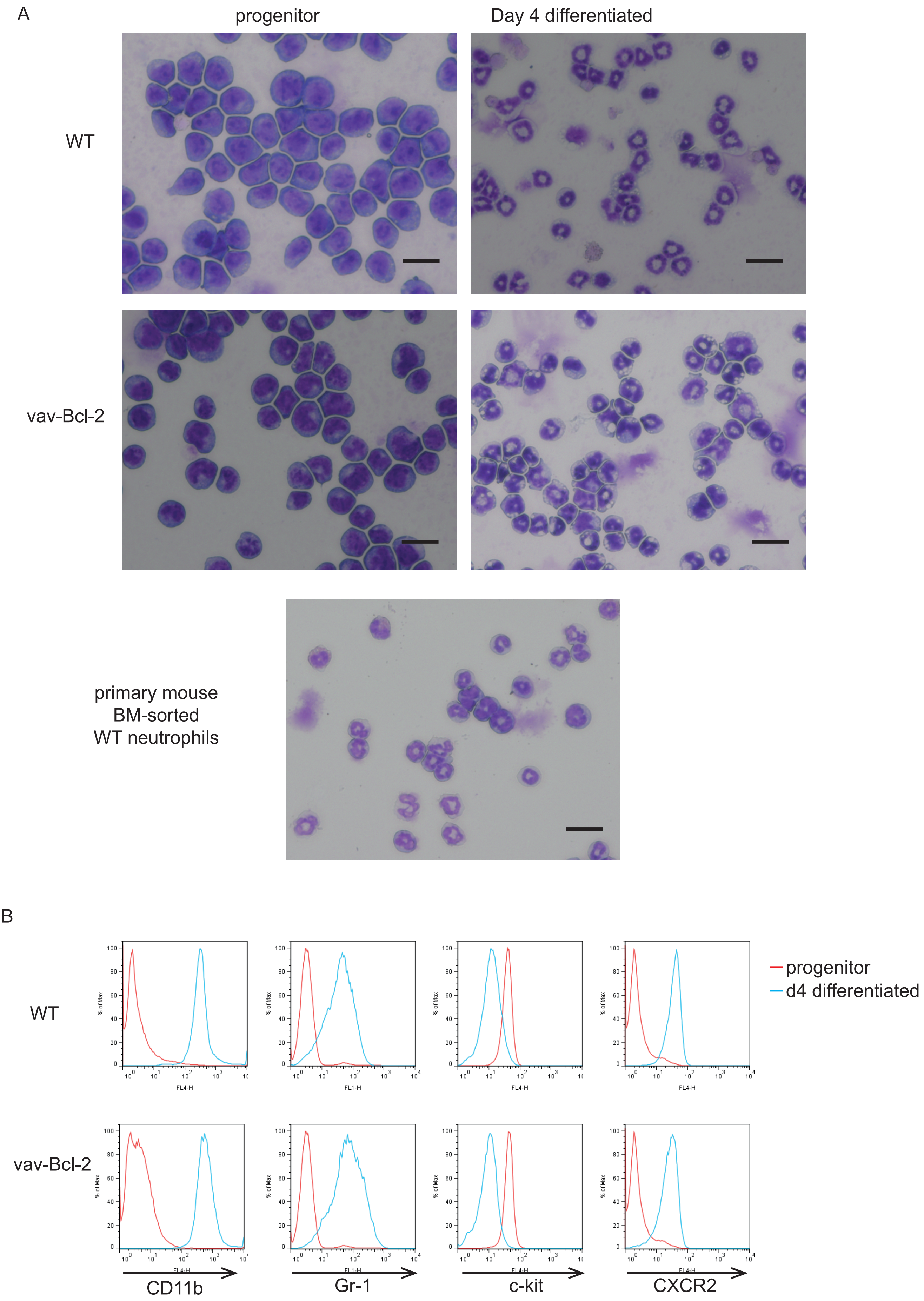
A
progenitor
Day 4 differentiated
WT
vav-Bcl-2
primary mouse
BM-sorted
WT neutrophils
B
WT
vav-Bcl-2
progenitor
d4 differentiated
CD11b
Gr-1
c-kit
CXCR2

Fig. S3

A

primary mouse BM-sorted neutrophils

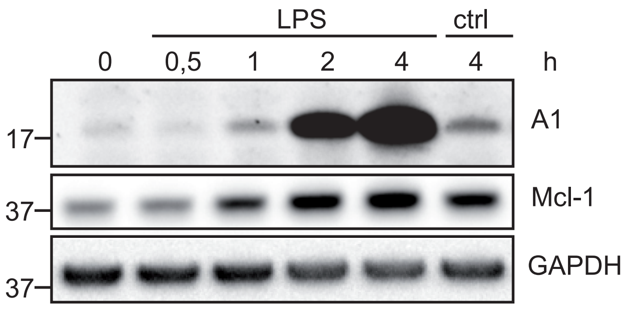

B

primary mouse BM-sorted neutrophils

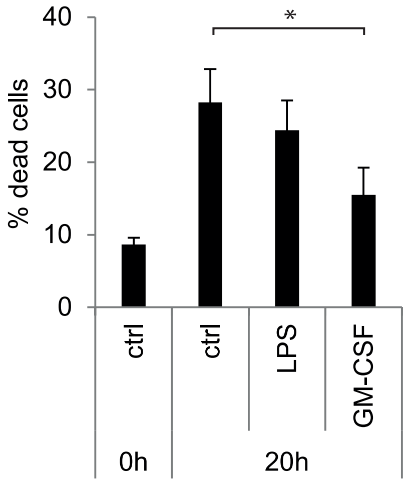

Fig. S4

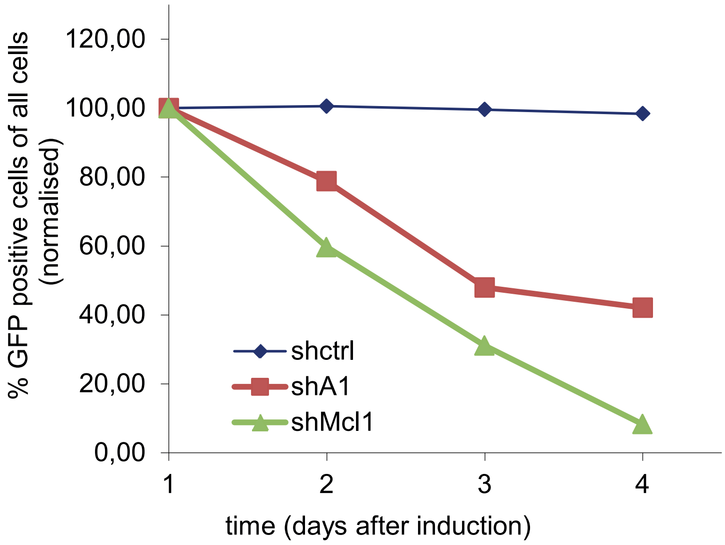

% GFP positive cells of all cells
(normalised)
120,00
100,00
80,00
60,00
40,00
20,00
0,00
1
2
3
4
time (days after induction)
shctrl
shA1
shMcl1

Fig. S5

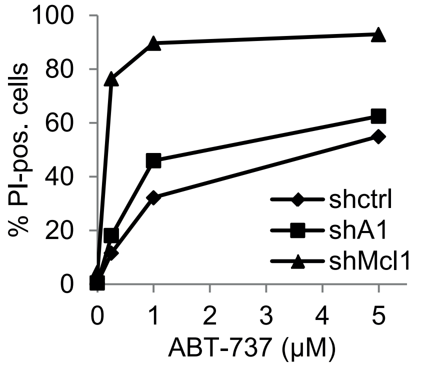
100
80
60
40
20
0
% PI-pos. cells
0
1
2
3
4
5
ABT-737 (µM)
shctrl
shA1
shMcl1

Fig. S6

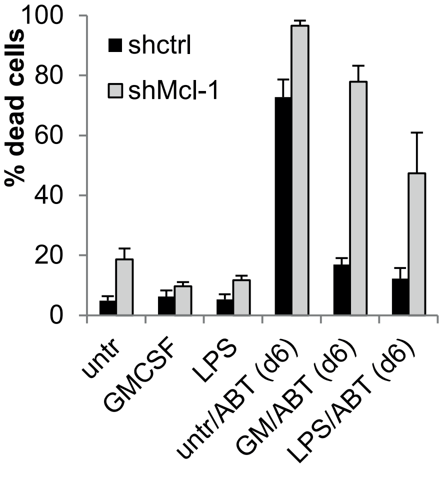
% dead cells
100
80
60
40
20
0
shctrl
shMcl-1
untr
GMCSF
LPS
untr/ABT (d6)
GM/ABT (d6)
LPS/ABT (d6)
